# Marbling Matters: Lean and Fatty Red Meat Show Opposing Associations with Brain Structural Indices

**DOI:** 10.3390/nu18101635

**Published:** 2026-05-21

**Authors:** Brandon S. Klinedinst, Alice L. Dawson, Michael DelCasale, Arjun Venkateswaran, Auriel A. Willette

**Affiliations:** 1 Department of Neurology, Robert Wood Johnson Medical School, Rutgers University, New Brunswick, NJ 08901, USA; brandon.klinedinst@rutgers.edu (B.S.K.); alice.dawson@rutgers.edu (A.L.D.); michaeldelcasale045@gmail.com (M.D.); arjunvenkateswaran329@gmail.com (A.V.); 2 Department of Nutritional Sciences, School of Environmental and Biological Sciences, Rutgers University, New Brunswick, NJ 08901, USA; 3 Institute for Health, Healthcare Policy, and Aging Research, Rutgers University, New Brunswick, NJ 08901, USA; 4 Brain Health Institute, Rutgers University, New Brunswick, NJ 08901, USA; 5 Krieger Klein Alzheimer’s Disease Center, Rutgers University, New Brunswick, NJ 08901, USA

**Keywords:** brain food, brain aging, UK Biobank, MRI, cortical thickness, white matter, gray matter, dietary epidemiology

## Abstract

**Background/Objectives:** Red meat is often treated as a single dietary category in nutritional epidemiology, despite substantial heterogeneity in fat content, quality parameters, and preparation methods. This may obscure meaningful associations with brain aging. We tested whether global brain structural associations differed across lean red meat, fatty red meat, pork, processed pork, and organ meat in a large community-based neuroimaging cohort. **Methods:** Participants were 45,811 UK Biobank adults aged 50 to 80 years with structural MRI, dietary recall, and covariate data. Dietary intake was assessed using up to five administrations of the Oxford WebQ 24 h recall and averaged across available timepoints. Global cortical thickness, total gray matter volume, and total white matter volume were derived from T1-weighted MRI. Continuous predictors were screened for linear quadratic, or spline form prior to grouped penalized variable selection. Final multivariable models incorporated sensitivity analyses stratified by socioeconomic status (SES) and sex. **Results:** Associations with global brain structure differed by meat type and fat content. Lean red meat showed the most favorable overall pattern, including modest nonlinear favorable association with global cortical thickness and a positive association with white matter volume among higher-SES participants. Fatty red meat showed unfavorable associations with cortical thickness and gray matter volume. Pork showed an unfavorable association with cortical thickness. Organ meat showed an unfavorable association with gray matter volume and with white matter volume among lower-SES participants. Overall, findings suggested that lean red meat tracked with neutral-to-favorable brain structural correlates, whereas fattier red meat and organ meat generally tracked with less favorable structural outcomes. **Conclusions:** Meat did not relate to global brain structure as a single uniform exposure. Instead, associations differed meaningfully by meat type, fat content, and socioeconomic context. Treating red meat as a single undifferentiated exposure may flatten biologically relevant heterogeneity and contribute to mixed prior findings. These results support more precise dietary phenotyping in brain-health research and suggest that distinctions in meat quality may matter when evaluating long-term brain aging. Findings should not be interpreted to suggest that unlimited meat intake is broadly health-promoting, even if lean, given the established cardiometabolic and vascular risks associated with excess intake of high-fat or processed meats.

## 1. Introduction

Brain health is an important endpoint in cognitive aging, as well as the aging of the peripheral nervous system. Alzheimer’s disease (AD), the leading cause of dementia, illustrates the scale of that challenge, with an estimated 7.2 million Americans are living with AD [1]. Structural brain differences and trajectories are detectable years to decades before clinical diagnosis, and neurodegeneration contributes to rising disability, loss of personal freedom, and healthcare costs. In recent decades, the field has rightly invested in genetic discovery and disease-modifying therapeutics. However, translation to population-wide prevention remains limited. Anti-amyloid monoclonal antibodies can modestly slow decline in selected early-stage patients, but eligibility, monitoring burden, cost, and safety considerations constrain their broad impact [2]. Meanwhile, the number of individuals living with AD continues to grow as populations age and real solutions have not yet emerged. These realities sharpen the case for prevention science: understanding modifiable exposures that accumulate across midlife and later life, including diet.

Diet is a biologically plausible upstream determinant of brain structure, because it shapes multiple pathways that converge on neural maintenance [3]. Some downstream pathways include cardiometabolic health and cerebral perfusion, gut microbiome-brain communication, lipid transport, astrocyte sheaths and other lipid neurobiology, inflammatory tone and oxidative stress, insulin signaling and glucose control, and synaptic plasticity. Recent work in nutritional neuroscience further supports the view that diet is linked to brain structure, function, and metabolism, and that healthier eating patterns tend to track with more favorable neuroimaging phenotypes, whereas less healthy patterns tend to track with poorer structural outcomes [4]. The nutrient matrices of foods function as upstream inputs [5]. Neural tissue is impacted by diet, implicating the infrastructure that undergirds cognition and whole-body organ function. As a result, analyzing food choice patterns alongside brain MRI phenotypes may capture cumulative biological “wear and tear”, offering both mechanistic insight and potential avenues for reducing the incidence of AD.

Amino acid metabolism is particularly relevant because amino acids contribute to neurotransmission, neuromodulation, energy metabolism, and nitrogen-containing compounds. Prior work suggests that amino acid profiles differ across cognitive status among older adults [6,7]. Because meat is a major dietary source of amino acids and differs substantially by fat content, processing, and food matrix, this literature supports the rationale for examining nutritionally distinct meat types in relation to MRI-derived markers of brain structure.

Because vascular and metabolic health are closely intertwined with brain aging [8,9], dietary factors that influence cardiometabolic risk may also affect neural integrity. Within this context, red meat is often treated as a single dietary category. It is commonly penalized in dietary scores because of concerns related to saturated fat and cardiometabolic disease. However, studies examining red meat in relation to cognition and brain-related outcomes have generally reported null or mixed findings [10]. A 2023 scoping review concluded that evidence linking red meat consumption with cognitive outcomes across the lifespan remains mixed, and that the topic is still relatively understudied [11].

A likely explanation for these inconsistent findings is that many studies aggregate nutritionally heterogenous meat exposures into a single “red meat” variable. If lean and fatty forms of red meat relate differently to brain aging, combining them may dilute or even cancel meaningful signal, creating the false appearance that no relationship exists. True patterns would be obscured by exposure misclassification [10,12]. This would explain the many null findings in prior literature. Supporting this possibility, a 2021 UK Biobank analysis found that processed meat intake was associated with greater risk of all-cause dementia, whereas unprocessed red meat intake was associated with lower risk, suggesting that important red meat heterogeneity may have meaningfully different associations with neurodegenerative outcomes [13].

Using UK Biobank, we analyzed approximately 45,000 participants with both structural MRI and 24 h dietary recall data. This large-scale community sample allowed stable estimation of associations between meat intake and a triangulation of global brain structure, including total gray matter volume, total white matter volume, and mean global cortical thickness, while also supporting sensitivity analyses that are often infeasible in smaller neuroimaging cohorts. It is important to note that UK Biobank participants have fewer health conditions and are more economically prosperous than the general population in the UK and former colonies or protectorates. Nonetheless, UK Biobank has mostly healthy volunteers, and exposure-outcome associations within the cohort remain informative for etiologic research when interpreted with appropriate caution. Further, the size and phenotypic depth of the cohort provide a unique opportunity to examine whether nutritionally distinct meat types show different relationships with broad structural brain MRI measures.

In the present study, we tested whether associations with global brain structure differed across lean red meat, fatty red meat, pork, and organ meat, rather than treating red meat as a single undifferentiated exposure. We further examined whether these associations remained evident in sensitivity analyses stratified by socioeconomic status and sex, given that food choice patterns, food quality, and broader dietary context may differ across these groups [14]. We hypothesized that separating meat type by fat content would reveal more informative and potentially divergent associations than conventional aggregate red meat measures, and that lean red meat would show a more favorable pattern of association with global brain metrics than fatty red meat.

## 2. Materials and Methods

### 2.1. Cohort Design

Participants were drawn from the UK Biobank, a large prospective cohort that enrolled approximately 500,000 adults recruited through 22 assessment centers across the United Kingdom beginning in 2006. Baseline assessments were completed between 2006 and 2010, and included the collection of dietary information. Each center visit followed a standardized sequence: (1) informed consent, (2) touchscreen questionnaire, (3) verbal interview, (4) ocular measurements, (5) physical measurements, and (6) collection of blood and urine samples. The touchscreen questionnaire captured sociodemographic characteristics and lifestyle factors. Brain MRI was acquired with scanning commencing in 2014. All participants provided written informed consent at baseline. UK Biobank received ethical approval from the North West—Haydock Research Ethics Committee.

The analytic sample was defined by the availability of (1) brain MRI data, (2) required demographic covariates, and (3) dietary recall data with a least one completed response out of the five possible assessments. Participants identified as extreme outliers based on calorie consumption were excluded (under 500 calories or over 6000 calories, per day). Applying these criteria yielded a final sample of 45,811 individuals. At the time of the commencement of the MRI scans, participants ranged from 50 to 80 years of age.

### 2.2. Global Brain Indices

A detailed description of UK Biobank MRI acquisition and processing procedures is publicly available in the UK Biobank imaging documentation (Resource 2367; http://biobank.ctsu.ox.ac.uk/crystal/refer.cgi?id=2367; accessed on 12 May 2026). Briefly, T1-weighted structural brain images were collected via a 3D MPRAGE sequence on a 3T Siemens MAGNETOM Skyra scanner (Siemens Healthineers, Erlangen, Germany), using a standard 32-channel RF head coil. Key information includes: sagittal acquisition; TR: 2000 ms; TE: 2.01 ms; TI: 880 ms; 1 mm^3^ isotropic voxels; flip angle: 8°; bandwidth: 240 Hz/Px; FOV: 256 mm; acceleration factor = 2. From these structural scans, we derived the global imaging phenotypes used in the present analyses: global cortical thickness, total gray matter volume, and total white matter volume. A FreeSurfer version 6.0 processing stream was used to derive these indices from the Desikan–Killiany atlas.

### 2.3. Food Intake Variables

Dietary intake was assessed using the UK Biobank online 24 h Dietary Recall Questionnaire (Oxford WebQ) [15]. The assessment was administered on up to five occasions approximately three to four months apart, to better approximate habitual intake. For each dietary variable, we calculated the mean across available administrations [16].

The Oxford WebQ (http://www.ceu.ox.ac.uk/research/oxford-webq, accessed on 12 May 2026) captures a broad range of foods and beverages consumed the previous day, including water, tea and coffee, alcoholic beverages, fruits and vegetables, whole and refined grains, dairy and eggs, fish and other seafood, meat, cooking oils and butter, and added salt. The questionnaire also provides an estimate of total energy intake (kcal/day), which was included as a modeled covariate.

To reduce dimensionality while preserving meaningful nutritional structure, some food variables were aggregated, and some were split using follow-up questions on food type or preparation. Our primary dietary exposures of interest here were red meat intake (beef, lamb), pork intake, processed pork intake (bacon, ham, sausage), and organ meat intake. A follow-up variable asking whether participants removed the fat from their red meat was used to route red meat intake into either lean red meat intake or fatty red meat intake.

This operational definition should be interpreted as a behavioral proxy for leaner versus fattier red meat intake rather than as a direct compositional measure of fat content. Participants were not asked to provide laboratory-based fat composition, cut-specific nutrient profiles, or objective measure of marbling, saturated fat, or cooking loss. Therefore, the lean/fatty distinction may contain exposure misclassification, which could attenuate true differences between leaner and fattier red meat or, alternatively, capture correlated behavioral factors such as food preparation practices, dietary restraint, or broader health consciousness. Accordingly, we interpret these variables as self-reported proxies rather than the objective biochemical composition of meat.

In the present analyses, tested dietary covariates included: refined grains (plain cereal, sweetened cereal, white pasta, white rice, refined baguettes, refined bread rolls, refined sliced bread), whole wheat grains (whole wheat cereal, bran cereal, whole wheat pasta, whole wheat baguettes, whole wheat bread rolls, whole wheat sliced bread), oats, brown rice, sweetcorn, Rosaceae pome fruits (apples, pears), Rosaceae stone fruits (cherries, peaches, plums, prunes), citrus fruits (oranges, orange juice, satsuma), grapefruit, Solanaceae fruits (tomatoes, sweet peppers), Cucurbitaceae fruits (cucumbers, zucchini), avocado, banana, mango, pineapple, olives, berries, grapes, butternut squash, melon, starchy legumes (baked beans, broad beans, green beans, peas, pulses), peanuts (salted and unsalted), Brassicaceae flower and leaf vegetables (broccoli, cauliflower, cabbage, kale, watercress, turnips), Apiaceae root and stalk vegetables (carrots, parsnips, celery), Amaryllidaceae bulbous vegetables (onions, garlic, leeks), Solanaceae tubers (boiled/baked potatoes, mashed potatoes, fried potatoes), nuts and seeds, lettuce, spinach, beetroot, mushrooms, sweet potatoes, whole milk, skim milk, plant milk, full-fat yogurt, reduced-fat yogurt, whole egg, skinless poultry, skin-on poultry, fish (white fish, tinned tuna, oily fish, battered fish, breaded fish), shellfish (shellfish, lobster, prawns), brewed coffee (espresso, filtered coffee, cappuccino, latte) instant coffee, green tea, black tea, red wine, white wine, fortified wine, beer/cider, spirits, drinking water, seed oil (sunflower oil, vegetable oil, other oil), rapeseed oil, butter, olive spread, butter spread, margarine, low-fat butter, lard, and olive oil.

### 2.4. Demographic and Covariate Variables

Socioeconomic status was operationalized using self-reported household income. The original five-level income variable was represented using four dichotomous indicators, with Middle Class income ($31,000 to $51,999) as the reference category: Under Class (less than $18,000), Lower Class ($18,000 and $30,999), Upper Class ($52,000 and $100,000), and High Class (greater than $100,000). For subgroup sensitivity analyses and interaction testing, income categories were collapsed into a binary SES variable to improve interpretability: Upper Class and High Class were combined as Higher SES, whereas Under Class, Lower Class, and Middle Class were combined as Lower SES.

Ethnicity was modeled using dichotomous indicators for White ethnicity and Indian ethnicity, with all other ethnicity categories serving as the omitted reference group. This coding reflected the distribution of the analytic sample, which was predominantly White, with smaller numbers in the other ethnicity categories. These terms were included for demographic covariate adjustment rather than for primary inference about ethnicity.

For highest level of educational obtainment, we created dichotomous indicators to contrast no credential obtainment with CSEs, GCSEs, A levels, professional training, or a university degree. Sex was modeled to contrast differences between women from men. For cannabis use, we created dichotomous indicators contrasting never/rare use with monthly, weekly, and daily use.

Additional covariates were modeled according to their observed scale. Age, physical activity, sleep duration, summertime outdoor exposure, medication burden, waist circumference, and intracranial volume were modeled as continuous variables. Tobacco exposure was represented by pack-years smoked and modeled continuously.

### 2.5. Winsorization of Predictor Variables

To reduce the influence of sparse upper-tail values while preserving the large mass of non-consumers, we winsorized selected quantitative food intake and covariate variables at the 95th percentile of the positive, non-missing observation. Values above this threshold were replaced with the 95th-percentile value, whereas zero values and missing values were left unchanged. After capping, these variables were rounded to the nearest whole number. Histograms were inspected before and after processing to confirm that the procedure reduced the impact of sparse extreme values without materially altering the broader distribution.

### 2.6. Determining the Functional Form of Continuous Predictors

Because the dietary exposure space was high-dimensional relative to the number of conceptually plausible food and lifestyle predictors, we used a staged analytic workflow designed to separate three decisions: functional-form specification, multivariable variable selection, and evaluation of subgroup heterogeneity. Functional forms were assessed before multivariable selection so that nonlinear exposure-outcome patterns would not be forced into linear terms. Penalized selection was then used to reduce the candidate predictor set while preserving grouped terms for quadratic and spline predictors. Finally, subgroup and interaction analyses were used to evaluate whether selected predictors showed evidence of differential associations across SES or sex. This sequence was intended to reduce arbitrary post hoc model-building by defining scale, shape, and inclusion of candidate predictors before final interpretation.

Before multivariate variable selection, we evaluated the functional form of each continuous lifestyle and dietary predictor in relation to the three global brain outcomes of interest. For each predictor for each brain outcome, we fit three separate regression specifications while adjusting for age, sex, and intracranial volume: a linear term, a quadratic term, and a natural cubic spline with 3 degrees of freedom. Additional curvature was assessed by nested model comparison using analysis of variance (ANOVA), and relative model fit was compared using Akaike’s Information Criterion (AIC) and Bayesian Information Criterion (BIC). In keeping with a parsimonious modeling strategy, linear specification was retained unless there was clear evidence that a quadratic or spline had materially improved data fit. Quadratic terms were preferred when a modest single-bend relationship appeared sufficient and scientifically interpretable. Spline terms were retained for variables with a wide continuous range and when the relationship appeared more complex, such as showing a threshold, leveling off across part of the exposure range, or otherwise departing from a simple linear or single-bend pattern. Functional forms were determined prior to variable selection and then carried forward into the penalized selection stage as grouped predictor blocks.

### 2.7. Variable Selection

Variable selection was conducted on the three global brain outcomes using a grouped penalized regression framework implemented with the grpreg package version 3.5.0 in R. Demographics were forced into all models and treated as unpenalized terms, including age, sex, socioeconomic status, educational attainment, ethnicity, and intracranial volume. Candidate lifestyle and dietary predictors, described in the preceding sections, were entered as penalized groups, with each group defined according to its prespecified functional form from the prior shape-selection step. Thus, predictors selected as linear were entered as a single coefficient, predictors selected as quadratic entered as a two-parameter block, and splines entered as a three-parameter block.

To perform selection, we applied group lasso over 200 random 50-50 split subsamples. Within each split, the penalty parameter was selected using AIC, and predictors were considered selected if any coefficient within their grouped block was non-zero at the chosen penalty value. For each candidate predictor, we then calculated the proportion of subsamples in which that predictor was selected. As shown in Supplementary Table S1, variables retained for the final multivariable model were those selected in at least 70% of the 200 split-half samples, reflecting a stability-based criterion intended to reduce overfitting and prioritize robust signal over single-sample fluctuation.

### 2.8. Sensitivity Analyses

To evaluate the robustness of diet, lifestyle, and covariate associations with each global brain outcome and to probe potential effect heterogeneity (for example, wealthier SES purchasing higher-quality food products) and distribution effects (for example, intake differences between men and women), we conducted prespecified subgroup sensitivity analyses stratified by SES and sex. We first fit an “overall” multivariate model in the full analytic sample using predictors carried forward from the variable selection stage. We then refit the same model structure within four subgroups: wealthier participants (‘High SES’), poorer participants (‘Low SES’), women, and men. These stratified models were used to identify predictors that demonstrated subgroup-specific evidence of association, particularly when a predictor was not statistically significant in the overall model but appeared statistically significant within one subgroup.

### 2.9. Final Model Specification

We then pooled subgroup findings in a single combined-sample model by evaluating interaction terms. For each predictor exhibiting evidence of subgroup-specific associations in stratified models, we refit the overall model including an interaction between that predictor and the relevant subgrouping variable (SES or sex), while retaining the corresponding main effects. Interaction terms were assessed as statistically significant at *p* < 0.05. Interaction terms that did not meet this criterion were instead treated as not demonstrating sufficient evidence of effect modification in the pooled analyses. Lastly, any terms not reaching at least trending significance (*p* < 0.10) were removed, producing the final models presented here.

After the stability-selection stage, retained predictors were entered into outcome-specific multivariable regression models. Subgroup analyses stratified by SES and sex were treated as sensitivity analyses intended to identify potential heterogeneity rather than as independent confirmatory tests. When a predictor showed subgroup-specific evidence of association, we evaluated the corresponding predictor-by-subgroup interaction in the pooled sample while retaining the relevant main effects. Interaction terms were interpreted as evidence of effect heterogeneity only when the interaction term reached *p* < 0.05 in the combined sample model. This final modeling approach was designed to prioritize predictors that demonstrated stability across repeated split-half samples and to avoid interpreting isolated subgroup findings without a corresponding pooled interaction test.

### 2.10. Figures

For significant red meat-brain associations retained in the final models, we generated model-implied prediction plots to visualize the estimated relationship between each meat intake variable and the relevant MRI-derived brain outcome. To aid interpretation, each significant association was displayed in two complementary forms. First, a full-range plot was generated using the observed outcome scale, with raw outcome values shown in the background and the model-implied fitted line overlaid. This version illustrates the modest magnitude of associations relative to the between-person variation in brain structure. Second, a magnified prediction plot was generated using the same model-implied fitted line, but with the *y*-axis restricted to the range of the model-implied predictions. This version was used to make the modeled shape, direction, and uncertainty of the association more visually interpretable. Shaded regions represent 95% confidence intervals.

### 2.11. Software

All data processing, statistical analyses, and figure generation were conducted in R version 4.5.1 (06-13-2025; R foundation for Statistical Computing, Vienna, Austria) on a 64-bit Windows platform. Reproducibility for procedures involving randomization or resampling was ensured by setting a fixed random seed (4201989).

### 2.12. AI-Assisted Editing Support

The authors used ChatGPT 5.5 Thinking Model, an AI-assisted writing tool, to assist with language refinement, clarity, and organization. AI assistance was limited to editorial and drafting support. All scientific content, analyses, interpretations, conclusions, and final wording were reviewed, verified, and approved by the authors, who remain fully responsible for the manuscript.

## 3. Results

As described in Table 1, the analytic sample included 45,811 participants with a mean age of 54 years (SD = 7.52); 52.1% were women and 60.3% were classified as lower SES. Mean global brain measures were 2.66 mm for cortical thickness, 660,489 mm^3^ for total gray matter volume, and 474,134 mm^3^ for total white matter volume.

**Figure 1:**
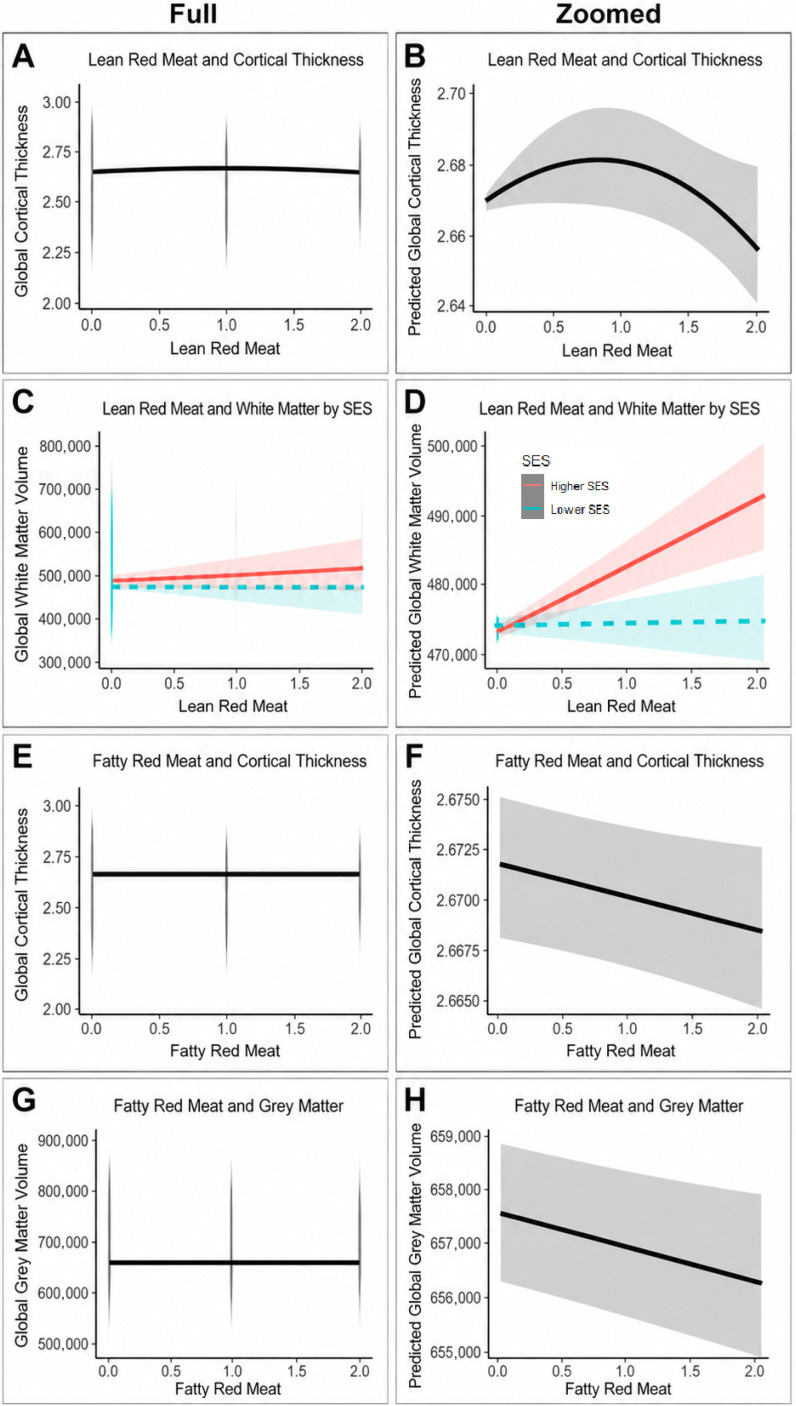
Model-implied prediction plots are shown for significant meat-brain associations. For each association, the left panel presents the full-range plot using the observed outcome scale, with the model-implied fit line overlaid. The right panel presents the corresponding magnified prediction plot, using the same model-implied fit line but restricting the *y*-axis to the range of model- implied predictions to clarify the modeled shape, direction, and uncertainty of the association. Shaded regions represent 95% confidence intervals. For SES-stratified panels, red solid lines indicate higher SES and teal dashed lines indicate lower SES.

**Figure 2:**
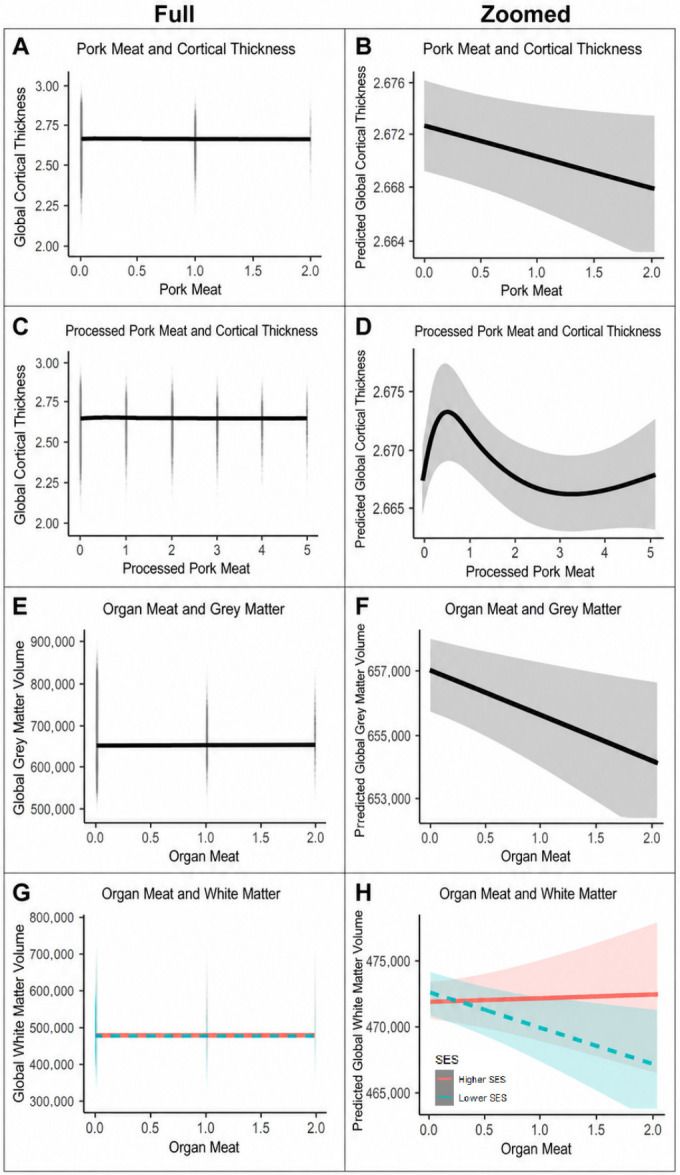
Model-implied prediction plots are shown for significant meat-brain associations. For each association, the left panel presents the full-range plot using the observed outcome scale, with the model-implied fit line overlaid. The right panel presents the corresponding magnified prediction plot, using the same model-implied fit line but restricting the *y*-axis to the range of model- implied predictions to clarify the modeled shape, direction, and uncertainty of the association. Shaded regions represent 95% confidence intervals. For SES-stratified panels, red solid lines indicate higher SES and teal dashed lines indicate lower SES.

Final multivariable model results are presented in Table 2, with corresponding visualizations of the meat-brain associations shown in Figure 1 and Figure 2. Associations between meat intake and global brain structure differed by meat type and, importantly, by fat content. Visual inspection of the fitted plots suggested that most meat-brain relationships were approximately linear, with only modest departures from linearity for selected exposures. By row for main effects of meat type/preparation, lean red meat showed modest positive linear and negative quadratic associations with global cortical thickness (Figure 1A,B), and a positive association with global white matter volume among higher-SES participants (Figure 1C,D). Fatty red meat showed negative linear associations with global cortical thickness (Figure 1E,F) and global gray matter volume (Figure 1G,H). Pork meat (Figure 2A,B) showed a negative linear association with global cortical thickness. Processed pork (Figure 2C,D) showed a modest non-linear pattern for cortical thickness. Lastly, organ meat demonstrated a negative linear association with gray matter (Figure 2E,F), and a negative linear association with white matter among lower-SES participants (Figure 2G,H).

While main effects for meat types/preparation styles were non-significant for global white matter, SES modified these relationships. For lean red meat, more consumption was related to more global white matter among Higher-SES groups but not Lower-SES groups. The inverse was seen with organ meat, where more consumption corresponded to less global white matter volume for only Low-SES groups. Together, these findings suggest that lean red meat intake tracked with either neutral or favorable brain structural correlates, whereas other meat types/preparation styles were generally related to less global parenchymal volume or cortical thickness.

Overall, the findings indicate that lean red meat showed more favorable associations with global brain structure than fatty red meat. The signal was not that “meat” moved in one direction as a single unanimous exposure, but rather that meat type, quality, and fat content mattered. In practical terms, these results suggest that treating all red meat as a single uniformly adverse exposure may flatten important biological distinctions.

## 4. Discussion

This large UK Biobank study found that associations between meat intake and global brain structure differed by fat content. Our findings suggest that leaner forms of red meat may have a more favorable association profile with global brain structure than fattier red meat. A positive association between white matter and lean red meat was observed among higher-SES participants and a modest nonlinear favorable pattern was observed for cortical thickness. Fatty red meat, pork, and organ meat showed less favorable associations. These findings suggest that treating red meat as a single undifferentiated exposure may obscure biologically meaningful heterogeneity. Rather, the nutritional distinction between leaner and fattier meat forms may be important for global brain health. The SES-stratified findings further suggest that the nutritional meaning of a food category may vary across socioeconomic contexts, potentially reflecting differences in food quality, preparation, and the broader dietary patterns in which meat is consumed. This has practical implications beyond academic classification; from a translational perspective, the results support a more nuanced research framework for evaluating meat consumption in relation to brain health. Our findings should not be interpreted as suggesting that high meat intake is broadly health-promoting, even if lean. Excess consumption of high-fat or processed meat remains well linked to adverse cardiometabolic and vascular outcomes, including higher LDL-related risk and greater risk of cardiovascular and cerebrovascular disease [17]. Rather, our results suggest that important differences between meat type and fat content may help inform more precise dietary decisions.

These results may help explain why prior studies linking red meat to cognition and neurodegenerative outcomes have often been null or mixed. If lean and fattier forms of red meat are combined into one exposure, opposing or divergent associations may cancel each other out, creating the impression that no clear relationship exists. Our findings are therefore consistent with the idea that nutritional heterogeneity within red meat categories contributes to inconsistent literature. More broadly, these findings suggest that future dietary frameworks may benefit from distinguishing food quality and preparation characteristics within broad meat categories, rather than treating all red meat exposures as nutritionally equivalent.

Several mechanisms could plausibly underlie the observed differences by meat type and fat content. Leaner and fattier meat forms are nutritionally distinct and may differ in their downstream cardiometabolic and inflammatory consequences. Fattier meat intake may reflect a greater very-long-chain saturated fat burden and a less favorable metabolic environment, as very-long-chain fatty acids depend on peroxisomal processing [18] and are linked to oxidative stress biology [19], while an excess of saturated fats more broadly can provoke lysosomal-mitochondrial injury pathways that elevate ROS production [20]. In contrast, leaner red meat may provide greater levels of protein and micronutrients, which are important for tissue upkeep while imposing a lower fat-related burden. The subgroup finding for white matter is particularly notable because white matter is lipid-rich and may be especially sensitive to metabolic and vascular conditions that support myelin integrity and structural connectivity. While these data do not permit causal or mechanistic inference, they are consistent with the broader hypothesis that meat quality and fat composition shape brain aging through intertwined nutritional, vascular, and neurobiological pathways.

The SES-stratified findings should be interpreted cautiously. Lean red meat was positively associated with white matter volume among higher-SES participants but not lower-SES participants, whereas organ meat was inversely associated with white matter volume among lower-SES participants. These patterns may reflect true effect modifications and may also reflect residual confounding by broader dietary quality, purchasing power, food preparation methods, access to different brands and meat cuts, or unmeasured lifestyle characteristics. For example, a self-reported “lean red meat” exposure may correspond to different food realities across socioeconomic strata, including differences in cut quality, trimming practices, cooking methods, or meal composition. Thus, the SES findings are best viewed as hypothesis-generating evidence that the nutritional meaning of a food category may vary across social context, rather than as definitive evidence that SES causally modifies the biological effect of meat intake.

Although the models adjusted for total energy intake and a broad set of dietary components, residual dietary confounding remains possible. Diet is not a collection of fully independent exposures. Foods may cluster into broader dietary patterns shaped by preference, culture, cost, access, health consciousness, and meal preparation. The favorable associations observed for lean red meat may partly reflect a broader dietary or lifestyle pattern rather than the effect of lean red meat by itself. The present findings should therefore be interpreted as adjusted associations within a complex dietary matrix, not as evidence that increasing lean red meat intake would improve brain structure.

While the observed associations were individually small, this is not unexpected for single dietary components evaluated alongside many correlated lifestyle, dietary, demographic, and anatomical covariates. These effect sizes should not always be interpreted as individually diagnostic or clinically actionable. Diet is a multidimensional composite exposure, and partitioning intake across 74 component variables necessarily distributes variance across many correlated behavioral inputs. An instructive parallel is physical activity: although physical activity as a whole may show a moderate association with health, that signal would likely fragment into smaller independent coefficients if it were decomposed into specific components such as walking, jogging, pushups, dips, squats, and related activities. The same logic applies to diet. Small associations may matter when exposures are common, repeated, and embedded within long-term dietary patterns, but the present cross-sectional estimates cannot determine whether such differences translate into meaningful longitudinal brain change or clinical risk.

These findings carry important implications for nutritional epidemiology and dietary assessment. Future dietary assessments may benefit from capturing an enhanced characterization of meat exposure, including quality-related features such as fat trimming practices, processing level, doneness, cooking method, conventional vs. organic, or grain-fed vs. grass-fed. Such detail may improve exposure precision and reduce misclassification. The findings also raise the possibility that dietary scoring indices which treat all red meat as uniformly adverse may be overly broad. Future work should move beyond broad meat categories toward scoring approaches that distinguish lean from fattier red meat and evaluate pork and organ meat separately, as these subtypes may differ meaningfully in their nutritional composition and health associations.

This study has several notable strengths, including the large UK Biobank imaging sample, repeated dietary assessment across five timepoints, simultaneous consideration of multiple meat subtypes, and triangulation across several global brain structural indices. The cohort size also permitted subgroup analyses that are often underpowered in smaller neuroimaging studies. Several limitations warrant consideration. The study is observational and cross-sectional because brain outcome measures were only included at baseline. Dietary intake was self-reported and is therefore vulnerable to recall and measurement error. The lean versus fatty distinction was not based on direct compositional assays, but rather it was defined by self-reported trimming/removal of visible fat, which is useful but imperfect. In addition, SES and ethnicity were represented using simplified categories. These variables were included to evaluate broad heterogeneity, but they do not capture the full social, cultural, or geographic complexity that may shape dietary intake and brain health. The UK Biobank healthy-volunteer bias can limit generalizability, particularly to more chronically ill populations. Finally, global MRI metrics may mask regional brain associations.

We prioritized global MRI indices because the purpose of this initial analysis was to test whether broad structural brain correlates differed across meat categories before moving to more anatomically granular hypotheses. Global cortical thickness, gray matter volume, and white matter volume provide summary measures of large-scale and divergent brain structure. However, this approach may mask region-specific associations including effects in brain regions that are differentially vulnerable to vascular, metabolic, or neurodegenerative processes. Future analyses should evaluate whether the observed global patterns are driven by specific cortical, subcortical, or white matter regions.

## 5. Conclusions

We sought to refine how red meat exposure is characterized in brain-health research by separating meat according to type and fat content. In summary, meat did not relate to global brain structure as a single uniform exposure. Instead, the findings point to meaningful heterogeneity among meat categories, with lean red meat showing a more favorable association profile than fatty red meat, pork, or organ meat. These results suggest that broad dietary narratives treating all red meat as nutritionally equivalent may obscure distinctions that matter for brain aging. Results should not be interpreted as causal evidence or as practical dietary guidance. Longitudinal studies, intervention designs, more objective dietary assessment, and region-specific neuroimaging analyses are needed to determine whether these associations reflect causal pathways, residual confounding, or broader dietary and socioeconomic patterning. More precise dietary phenotyping may help strengthen future research, improve exposure measurement, and support dietary guidelines that better reflect the biological complexity of real foods.

## Figures and Tables

**Table 1:** High class and Upper class were aggregated to form High SES, and Under class, Lower class, and Middle class were aggregated to form Lower SES. Categorical variables are expressed as count (percentages). Continuous variables are expressed as mean (standard deviation).

Characteristic	Summary
**Demographics and Socioeconomic Factors**	
Age (years)	54.48 (7.52)
Female	23,875 (52.1%)
Male	21,936 (47.9%)
Higher SES:	18,196 (39.7%)
Upper class ($52,000 to $100,000)	13,774 (30.1%)
High class (over $100,000)	4422 (9.7%)
Lower SES:	27,615 (60.3%)
Underclass (under $18,000)	4299 (9.4%)
Lower class ($18,000 to $30,999)	9561 (20.9%)
Middle class ($31,000 to $51,999)	13,755 (30.0%)
Education, CSE	944 (2.1%)
Education, GCSE	4292 (9.4%)
Education, A-level	7777 (17.0%)
Education, Professional	6972 (15.2%)
Education, College Degree	24,099 (52.6%)
Ethnicity:	
White Ethnicity	44,288 (96.7%)
Indian Ethnicity	411 (0.9%)
Other Ethnicity	1112 (2.4%)
**Lifestyle and Health Factors**	
Physical Activity (minutes/week)	122.29 (71.07)
Sleep Duration (hours)	7.17 (0.84)
Never Used Tobacco	33,364 (72.8%)
Tobacco (pack-years)	4.47 (9.58)
Cannabis Use:	
Never/Rare Cannabis Use	42,861 (93.6%)
Daily Cannabis Use	507 (1.1%)
Weekly Cannabis Use	1389 (3.0%)
Monthly Cannabis Use	1054 (2.3%)
Summertime Outdoors (hours)	3.49 (1.68)
Medication Count	1.78 (1.71)
Waist Circumference (cm)	88.21 (11.98)
**Brain Measures**	
Intracranial Volume (mm^3^)	1,546,656 (153,361)
Global Gray Matter Volume (mm^3^)	660,489 (59,343)
Global White Matter Volume (mm^3^)	474,134 (57,303)
Global Cortical Thickness (mm)	2.66 (0.11)
**Meat Intake**	
Lean Red Meat (servings/day)	0.01 (0.12)
Fatty Red Meat (servings/day)	0.44 (0.62)
Pork (servings/day)	0.14 (0.38)
Processed Pork (servings/day)	1.03 (1.41)
Organ Meat (servings/day)	0.03 (0.20)

**Table 2:** Standardized regression coefficients (β) and *p*-values from final multivariable models predicting global cortical thickness, total gray matter volume, and total white matter volume. Only predictors retained in the final models are shown; blank cells indicate that a predictor was not retained for that outcome. L denotes the linear component and Q denotes the quadratic component. Spline 1–3 denotes basis coefficients from natural cubic spline terms and should be interpreted jointly as representing a nonlinear association. For predictors with evidence of socioeconomic status (SES) interaction, stratum-specific coefficients are shown. Sex coefficients are shown relative to the female reference group, and ethnicity coefficients are shown relative the other omitted reference ethnicity groups.

	Predictor	Cortical Thickness	Gray Matter Volume	White Matter Volume
**Covariates**	AgeT1	β = −0.351 (*p* < 0.001)	β = −0.224 (*p* < 0.001)	β = −0.164 (*p* < 0.001)
	Sex	Male: β = −0.052 (*p* < 0.001)	Male: β = 0.113 (*p* < 0.001)	Male: β = 0.064 (*p* < 0.001)
	White Ethnicity	β = 0.012 (*p* = 0.017)	β = 0.034 (*p* < 0.001)	
	Indian Ethnicity	β = 0.002 (*p* = 0.636)	β = −0.009 (*p* = 0.001)	
	Physical Activity	L: β = 0.040 (*p* = 0.021) Q: β = −0.024 (*p* = 0.158)	Spline: 1: β = 0.007 (*p* = 0.003) 2: β = 0.009 (*p* = 0.001) 3: β = 0.005 (*p* = 0.083)	L: β = 0.028 (*p* = 0.003) Q: β = −0.014 (*p* = 0.134)
	Sleep Duration	L: β = 0.113 (*p* = 0.009) Q: β = −0.137 (*p* = 0.001)	L: β = 0.110 (*p* < 0.001) Q: β = −0.118 (*p* < 0.001)	
	Tobacco Packyears	β = −0.025 (*p* < 0.001)	β = −0.014 (*p* < 0.001)	L: β = 0.011 (*p* = 0.113) Q: β = −0.024 (*p* = 0.001)
	Daily Cannabis		β = 0.005 (*p* = 0.034)	
	Weekly Cannabis			
	Monthly Cannabis			
	Summertime Outdoors	Spline: 1: β = −0.010 (*p* = 0.052) 2: β = 0.001 (*p* = 0.830) 3: β = −0.022 (*p* < 0.001)	Spline: 1: β = −0.005 (*p* = 0.088) 2: β = 0.002 (*p* = 0.538) 3: β = −0.008 (*p* = 0.012)	L: β = 0.013 (*p* = 0.226) Q: β = −0.006 (*p* = 0.567)
	Medication Count	β = −0.040 (*p* < 0.001)	β = −0.030 (*p* < 0.001)	β = −0.014 (*p* < 0.001)
	Waist Circumference	L: β = 0.311 (*p* < 0.001) Q: β = −0.292 (*p* < 0.001)	Spline: 1: β = −0.006 (*p* = 0.122) 2: β = −0.002 (*p* = 0.656) 3: β = −0.023 (*p* < 0.001)	L: β = 0.075 (*p* = 0.005) Q: β = −0.126 (*p* < 0.001)
	Intracranial Volume	β = 0.030 (*p* < 0.001)	β = 0.760 (*p* < 0.001)	β = 0.819 (*p* < 0.001)
	Energy Intake		β = 0.015 (*p* < 0.001)	
**Meats**	Lean Red Meat	L: β = 0.029 (*p* = 0.049) Q: β = −0.031 (*p* = 0.037)		Low SES: β = 0.000 (*p* = 0.740) High SES: β = 0.011 (*p* < 0.001)
	Fatty Red Meat	β = −0.009 (*p* = 0.033)	β = −0.005 (*p* = 0.033)	
	Pork	β = −0.008 (*p* = 0.046)		
	Processed Pork	Spline: 1: β = −0.010 (*p* = 0.034) 2: β = 0.013 (*p* = 0.013) 3: β = −0.007 (*p* = 0.119)		
	Organ Meat		β = −0.004 (*p* = 0.045)	Low SES: β = −0.006 (*p* = 0.009) High SES: β = 0.000 (*p* = 0.927)

## Data Availability

The data analyzed in this study were obtained from UK Biobank under Application Number 343478. UK Biobank data are not publicly available without registration and approval, but are available to bona fide researchers for health-related research in the public interest through the UK Biobank Access Management System.

## Supplementary Materials

The following supporting information can be downloaded at: https://www.mdpi.com/article/10.3390/nu18101635/s1, Table S1: Selected Predictors.
