# Supplementary material for: Marbling Matters: Lean and Fatty Red Meat Show Opposing Associations with Brain Structural Indices
Source: Nutrients. 2026 May 21;18(10):1635. doi: 10.3390/nu18101635 (PMC13209396; doi:10.3390/nu18101635)
Supplement: Supplementary file 1 [file nutrients-18-01635-s001.zip › nutrients-4278474-supplementary.pdf]

Supplementary Table S1. Selected Predictors

Predictor	Global Grey Matter	Global White Matter	Global Cortical Thickness
**Physical Activity**	**1.0**	**1.0**	**.96**
**Sleep Duration**	**1.0**	**1.0**	**1.0**
**Tobacco Packyears**	**1.0**	**1.0**	**1.0**
**Daily Cannabis**	**.765**	.36	**.78**
**Weekly Cannabis**	.28	.49	.36
**Monthly Cannabis**	.365	.395	.285
**Summertime Outdoors**	**.995**	**.965**	**1.0**
**Medication Count**	**1.0**	**1.0**	**1.0**
**Waist Circumference**	**1.0**	**1.0**	**1.0**
**Energy Intake**	**1.0**	.53	.24
**Drinking Water**	.5	.34	**1.0**
**Brown Rice**	.235	.405	**.715**
**Refined Grains**	**.955**	**.81**	**.8**
**Whole Wheat**	**.965**	**.915**	.64
**Oats**	**.98**	**.695**	**.765**
**Fortified Wine**	.46	.615	.37
**Rosaceae Pome**	**1.0**	.475	**.845**
**Rosaceae Stone**	.31	.4	.325
**Citrus**	.67	**.92**	.26
**Grapefruit**	.49	.67	**.795**
**Starchy Legumes**	.635	**.995**	.39
**Peanuts**	.335	**.71**	.505
**Brassicaceae**	**.885**	.455	.385
**Apiaceae**	.65	.555	**.895**
**Allium**	.485	**.945**	.27
**Solanaceae**	**.895**	.29	**.795**
**Cucurbitaceae**	.25	.41	.47
**Potatoes**	**.865**	**.775**	.365
**Nuts and Seeds**	**.735**	.445	.25
**Avocado**	.33	.385	.435
**Banana**	**.73**	.3	.415
**Mango**	.525	.575	.335
**Pineapple**	.42	**.84**	.355
**Olives**	.355	.415	.47
**Berries**	**.92**	.425	.405
**Grapes**	.625	.67	**.86**
**Squash**	.38	**.7**	.48
**Melon**	.345	.265	.345
**Lettuce**	.645	**.79**	.295
**Spinach**	**.76**	**.9**	.395
**Beetroot**	.305	**.72**	.31
**Mushrooms**	**.725**	.505	.35
**Sweet Corn**	**.835**	.43	**.87**
**Sweet Potato**	.305	.375	.325

**Brewed Coffee**	.51	**.835**	**.91**
**Instant Coffee**	.655	**.88**	**1.0**
**Green Tea**	.355	.405	.585
**Black Tea**	**.995**	**.895**	**1.0**
**Red Wine**	**.98**	**.72**	**1.0**
**White Wine**	**1.0**	**.95**	**.965**
**Beer or Cider**	.48	**1.0**	**.975**
**Spirits**	**.87**	.53	**.715**
**Soda Pop**	**1.0**	.42	**.84**
**Hard Cheese**	**.895**	**.78**	.355
**Low Fat Hard Cheese**	**.91**	**.76**	.605
**Soft Cheese**	**.955**	**.99**	**.765**
**Eggs**	.485	**.73**	.44
**Reduced Fat Yogurt**	.435	**.86**	.36
**Full Fat Yogurt**	**.79**	**.9**	**.945**
**Skim Milk**	.29	.34	.4
**Dairy Fat Milk**	.425	.39	.505
**Poultry**	.535	**1.0**	.29
**Skinless Poultry**	.515	.59	.655
**Fish**	.28	.57	.635
**Shellfish**	**.835**	.595	.28
**Lean Red Meat**	**.83**	**1.0**	**.945**
**Fatty Red Meat**	**.94**	.315	**.895**
**Pork**	**.805**	.285	**.785**
**Processed Pork**	.385	.23	**.97**
**Organ Meat**	**.875**	**.86**	.66
**Salt Added**	.38	**.99**	**.985**
**Olive Oil**	.23	.32	.635
**Vegetable Oil**	.285	**.715**	.52
**Rapeseed Oil**	**.81**	.495	.55
**Low Fat Butter**	.595	.395	.29
**Butter**	.275	**.77**	.335
**Lard**	.38	.3	.325
**Olive Oil Spread**	.42	.665	.475
**Butter Spread**	.31	.56	.315
**Margarine**	.305	**.955**	.33

Supplementary Table S1. Values represent the proportion of 200 random split-half subsamples in which each candidate predictor was selected by the group penalized regression procedure for the corresponding brain outcome. Values closer to 1.0 represent more consistent selection across subsamples. Predictors selected in at least 70% of subsamples were retained for sensitivity analyses and the final multivariable models.
